# Hg isotopic composition and total Hg mass fraction in NIES Certified Reference Material No. 28 Urban Aerosols

**DOI:** 10.1007/s00216-020-02691-9

**Published:** 2020-05-18

**Authors:** Akane Yamakawa, Sylvain Bérail, David Amouroux, Emmanuel Tessier, Julien Barre, Tomoharu Sano, Kimiyo Nagano, Sadia Kanwal, Jun Yoshinaga, Olivier F. X. Donard

**Affiliations:** 1 Center for Environmental Measurement and Analysis, National Institute for Environmental Studies (NIES), 16-2 Onogawa, Tsukuba, Ibaraki, 305-8506 Japan; 2 Universite de Pau et des Pays de l’Adour, E2S UPPA, CNRS, IPREM, Institut des Sciences Analytiques et de Physicochimie pour l’Environnement et les Matériaux, 2 Avenue Pierre Angot, 64053 Pau Cedex 09, France; 3 Advanced Isotopic Analysis, Technopole Hélioparc Pau Pyrénées, 2 Avenue Pierre Angot, 64053 Pau Cedex 09, France; 4 Graduate School of Frontier Sciences, The University of Tokyo, 5-1-5 Kashiwanoha, Kashiwa, Chiba 277-8563 Japan; 5 Faculty of Life Sciences, Toyo University, 1-1-1 Izumino, Itakura, Oura, Gunma 394-0193 Japan

**Keywords:** Mercury, Particulate matter, CRM, Quality assurance/quality control, THg, Hg isotope

## Abstract

An interlaboratory study on the National Institute for Environmental Studies (NIES) certified reference material (CRM) No. 28 Urban Aerosols, collected on the filters of a central ventilating system in a building in Beijing city center, was performed to obtain informative values of Hg isotopic composition and total Hg (THg) mass fraction. The THg mass fraction was determined by four organizations using atomic absorption spectrometry; it resulted in the mean value of 1.19 ± 0.12 mg/kg (2SD, *n* = 24). The Hg isotopic composition of the CRM was measured and intercompared at two different institutions by cold vapor generation system coupled to multicollector inductively coupled plasma mass spectrometry. Subsequently, a conventional dissolution method that uses a mixture of HNO_3_/HCl/H_2_O_2_ in Hotblock® and two different dissolution methods that use a mixture of HNO_3_/HCl with a microwave and a digestion bomb were applied. The Hg isotopic compositions were δ^202^Hg = − 1.26 ± 0.17‰, Δ^199^Hg = − 0.23 ± 0.06‰, Δ^200^Hg = 0.01 ± 0.07‰, and Δ^201^Hg = − 0.22 ± 0.09‰ (2SD, *n* = 18) for the conventional method, which agree well with those obtained using microwave and bomb digestion. Our results indicate that, for the quality control of particulate matter analyses, this CRM is appropriate for use in environmental and geochemical studies.

**Electronic supplementary material:**

The online version of this article (10.1007/s00216-020-02691-9) contains supplementary material, which is available to authorized users.

**Figure:**
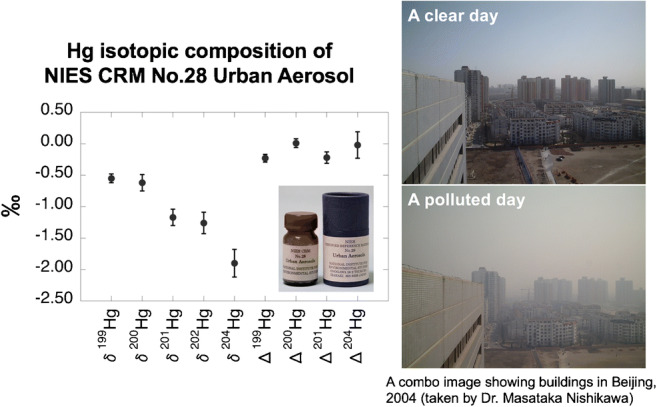
Graphical abstract

## Introduction

Anthropogenic emissions of particulate matters from urban and industrial areas are a critical environmental problem. High concentrations of particulate matter (PM) emissions are attributed to combustion from multiple sources, such as household fuel use, industrial activities, open burnings (such as agricultural and e-waste burnings), and transportation-related emissions. After an instance of extremely severe pollution occurred in 2011 in China, PM2.5 pollution has attracted considerable attention not only from scientists but also from the general public and from governments around the world. PM is a compound pollutant characterized by a complex chemical composition. To provide a scientific view for improving air quality, identifying the source(s) of the emitted PMs is of great importance. The results of chemical analyses performed on PMs provide an indication of the identity of the source(s). For example, the presence of K in aerosol samples can be attributed to biomass emissions [1]; that of Si and Ca can be attributed to soil, road, and construction dust; Fe, Mn, and Cu found together can be attributed to minerals used in industrial productions and processes [2]; and the presence of Pb can be associated with emissions from motor vehicles [3]. Hg, which has caused a great deal of concern due to the toxicity of its methylated derivative, is also known to be emitted as a result of coal combustion and the production of cement or steel [4]. Hg exists in three chemical forms in the atmosphere. The dominant form (> 95% of the total amount of Hg in the lower atmosphere) is the gaseous elemental Hg (GEM, Hg^0^_(g)_). The other two chemical species, gaseous oxidized Hg (GOM, Hg^2+^_(g)_) and particulate-bound Hg (PBM, Hg_(p)_), are relatively reactive and are efficiently removed from the atmosphere through wet and dry depositions. The atmospheric residence time of GEM is relatively long (~ 1 year) and allows regional and global transportation from emission sources. During such transportation, GEM is converted to GOM and PBM via atmospheric reactions. PBM can be derived from direct anthropogenic sources or produced via atmospheric transformations, and depleted by either photoreduction or deposition. The global median values at rural sites and high-elevation sites were 4.6–11.0 pg/m^3^ [5]; however, Beijing, a megacity, demonstrated considerably higher Hg mass fractions in the collected PM (263 ± 246 pg/m^3^ at daytime and 280 ± 383 pg/m^3^ at nighttime [6]).

Based on measurement data, alternative approaches help to understand the nature of the source/transport/receptor relationships, and provide accurate assessment of air quality problems as well as the most effective and efficient approaches to improve the air quality. The Tekran^®^ 2537/1130/1135 system (Tekran Instrument Corp., Canada) has been widely used to measure atmospheric Hg under various environments. The Tekran^®^ 2537 module measures GEM or TGM (total gaseous Hg, TGM = GEM + GOM), and the 1130 and 1135 components measure GOM and PBM, respectively. This instrument has high temporal resolution (typically, 5 min for GEM and 1–2 h for GOM and PBM). Recent advancements in stable Hg isotope analysis provide unique insights into the sources (anthropogenic vs. natural) and processes of Hg in the environment. Mass-dependent fractionation (MDF) and mass-independent fraction (MIF) of Hg isotopes were observed in geological and biological materials (summalized in Blum et al. [7]). The MDF of Hg isotopes can be induced by many natural processes (such as reduction and oxidation, methylation and demethylation, sorption, evaporation, and volatilization), whereas the large MIF of Hg isotopes is primarily produced by photochemical reactions (discussed in detail in Blum et al. [7]). Previously, studies suggest that determining Hg isotope ratios may be used to distinguish between background atmospheric pool and emission sources [8–11]. Xu et al. demonstrated that the values of δ^202^Hg and Δ^199^Hg in PM2.5 were different among aerosol samples collected in three cities in China (Beijing, Changchun, and Chengdu) [12]. According to their data, the mean δ^202^Hg value in PM2.5 became higher in the order of Changchun, Beijing, and Chengdu, and the variation trend of Δ^199^Hg was decoupled from that of δ^202^Hg. These results indicate that the relative contributions of the emission sources might significantly differ between provinces or cities.

Combining these different strands of evidence would provide a better view and understanding of the complex issue of the origin and fate of PM in the environment. To identify the emission source(s) of PMs, an appropriate certified reference material (CRM) that provides the means for obtaining accurate analytical data is necessary. Aerosol CRMs for elemental analysis have been produced by the National Institute for Environmental Studies (NIES, CRM No. 28 Urban Aerosols), the National Institute of Standards and Technology (NIST, SRM 1648a Urban Particulate Matter), and the European Commission Joint Research Centre–Institute for Reference Materials and Measurements (JRC–IPMM, ERM-CZ120 Fine Dust (PM10-like)). The Hg mass fraction has been determined for NIST SRM 1648a (1.323 ± 0.064 mg/kg), but Hg isotopic compositions have not been reported yet for any urban particulate reference material. In this study, NIES CRM No. 28, collected in Beijing, was selected to determine the Hg isotopic reference values of aerosol referenced materials. Because south and north Asia are areas of major concern in terms of the atmospheric pollution problem, NIES CRM No. 28 will be an appropriate CRM to use when attempting to identify the sources of PM emissions.

As an interlaboratory study on the CRM, isotopic composition was measured at the NIES and at the Institut des Sciences Analytiques et de Physico-chimie pour l’Environnement et les Matériaux (IPREM) using cold vapor generation coupled to multicollector inductively coupled plasma mass spectrometry (CV-MC-ICP-MS). Moreover, the total Hg (THg) mass fraction was also determined by four organizations using atomic absorption spectrometry.

## Materials and methods

### NIES CRM No. 28 Urban Aerosols

NIES CRM No. 28 Urban Aerosols was produced to evaluate the analytical accuracy of the determination of the mass fraction of selected elements. Material preparation, analytical protocols, and 18 certified and 14 reference values, expressed as mass fraction [13], have been reported [14] (Table 1). The original PM was collected from the filters of a central ventilating system of a building located in Beijing city center from 1996 to 2005. The PM was recovered from the filters by mechanical vibration and sieved to remove coarse particles. The diameters of the particles were measured, and the results indicated that 99% of them were < 10 μm. Thus, 2 kg of the collected PM was subdivided into 1031 prewashed amber glass bottles. Then, multi-element analysis was performed on 12 bottles randomly selected among the mentioned 1031 bottles. The values for the between- and within-bottle standard deviations for each element were < 3%; therefore, the evidence confirmed that the prepared material was sufficiently homogeneous to be used as a CRM.

**Table 1:** Certified and reference values of NIES CRM No. 28 Urban Aerosols

	Element	Unit	Mass fraction
Certified values	Na	%	0.796 ± 0.065
	Mg	%	1.40 ± 0.06
	Al	%	5.04 ± 0.10
	K	%	1.37 ± 0.06
	Ca	%	6.69 ± 0.24
	Ti	%	0.292 ± 0.033
	Fe	%	2.92 ± 0.17
	Zn	%	0.114 ± 0.010
	V	mg/kg	73.2 ± 7.0
	Mn	mg/kg	686 ± 42
	Ni	mg/kg	63.8 ± 3.4
	Cu	mg/kg	104 ± 12
	As	mg/kg	90.2 ± 10.7
	Sr	mg/kg	469 ± 16
	Cd	mg/kg	5.60 ± 0.43
	Ba	mg/kg	874 ± 65
	Pb	mg/kg	403 ± 32
	U	mg/kg	4.33 ± 0.26
Reference values	Si	%	14.9
	P	%	0.145
	S	%	3.91
	Cl	%	0.807
	Sc	mg/kg	10.7
	Co	mg/kg	22.0
	Se	mg/kg	14.4
	Rb	mg/kg	64.1
	Y	mg/kg	21.9
	Mo	mg/kg	28.4
	Sn	mg/kg	21.5
	Sb	mg/kg	20.1
	La	mg/kg	32.7
	Th	mg/kg	11.1

### Reagents for Hg isotopic analysis

NIST SRM 3133 (an Hg isotopic standard solution) was used as primary standard, and NIST SRM 997 (thallium isotopic standard solution) was used to conduct an internal mass bias correction for the Hg isotope analysis. To ensure the validity of the analyses, NIST RM 8610 (UM Almaden, Hg isotopic standard solution) and BCR-176R (Fly Ash) were used as secondary standards.

#### IPREM

HCl and HNO_3_ for sample preparation and dilutions were BAKER INSTRA-ANALYZED Reagent (JT Baker, USA), and H_2_O_2_ was an optima grade from Fisher Chemical (UK). Ultrapure HNO_3_ (Ultrex II grade, JT Baker, USA) was used for Tl solution preparation. Tin (II) chloride dihydrate (SnCl_2_·2H_2_O) was of reagent grade from Scharlau (Spain). All aqueous solutions were prepared using ultrapure water (18 MΩ, Millipore, USA).

#### NIES

Ultrapure-100 grade HCl and HNO_3_, and reagent-grade tin (II) chloride dihydrate (SnCl_2_·2H_2_O) were purchased from Kanto Chemical Co., Inc. (Japan). All aqueous solutions were prepared using ultrapure water (18 MΩ, Millipore, USA).

### Sample preparation

As the between-bottle and within-bottle homogeneities of Hg have not been verified, we randomly selected four bottles for analysis to assess between-bottle variation. Note that ~ 0.3 g of the three subsamples of CRM (bottle no. 581, 901, and 990) was decomposed using HNO_3_, HCl, and H_2_O_2_ in HotBlock® (TJ Environmental, The Netherlands), which was maintained at 85 °C for 24 h (v/v = 3:1:1), using the method described in Foucher et al. [15] and Guedron et al. [16]. Moreover, we used two different digestion methods to ensure the stability of analytical values. Three subsamples (0.05 g for each) collected from bottle no. 960 were digested for 3 h by HNO_3_ and HCl (v/v = 1:3) in a digestion bomb that was maintained at 130 °C at the University of Tokyo. Three subsamples, weighing ~ 0.3 g each, were collected from each of two bottles (no. 581 and 990) and digested by HNO_3_ and HCl (v/v = 3:1) using a microwave system (UltraWave, Milestone, Italy) at 230 °C for 25 min at IPREM. To manage the analytical accuracy of our method, Hg isotopic measurement of the secondary reference, BCR-176R Fly Ash [17], was performed using the same methods. Hg mass fractions of all dissolved samples were then measured using CV-MC-ICP-MS by sample-standard bracketing method and adjusted to 0.5 and 1.0 ng/mL at IPREM and NIES, respectively. The acid composition of the final solutions are 10% HNO_3_ and 2% HCl (v/v) for IPREM and 2% HNO_3_ and 8% HCl (v/v) for NIES. Note that, to monitor the instrument’s stability, Hg isotopic composition was analyzed at least twice on different days.

### Hg isotopic measurements

Hg isotopic measurements were conducted by CV-MC-ICP-MS, using a Nu Plasma II instrument at NIES and a Nu Plasma instrument at IPREM (both from Nu Instruments, UK). In particular, Nu Plasma II was interfaced with an Aridus II desolvating nebulizer for Tl introduction and an HGX-200 cold vapor (CV) system (both from Teledyne CETAC Technologies, USA) for Hg^0^ generation at NIES, whereas Nu Plasma was interfaced with a DSN-100 desolvating nebulizer (Nu Instruments, UK) and a home-made CV system at IPREM. Hg^0^ and Tl dry aerosols (introduced Tl concentration = 15 μg/L) were mixed at the outlet of the CV generation system before they were introduced into the plasma. Sample and standard solutions were diluted to appropriate Hg and acid concentrations (~ 10% HNO_3_ and ~ 2% HCl, v/v), and Hg^2+^ was reduced online with 3% SnCl_2_ in ~ 10% HCl. Hg and Tl isotopes were monitored simultaneously, and a value of 2.38714 for the ^205^Tl/^203^Tl isotope ratio for NIST SRM 997 was used for instrumental mass bias correction applying an exponential law.

In this study, the mass numbers of 198 (Hg), 199 (Hg), 200 (Hg), 201 (Hg), 202 (Hg), 203 (Tl), 204 (Hg, Pb), 205 (Tl), and 206 (Pb) were detected by individual Faraday cups. The preamplifier gains associated with each Faraday cup were calibrated daily. Instrumental parameters were then tuned each day prior to the analysis in order to obtain maximum signal intensity and stability.

#### Details of the procedure implemented at IPREM

From each sample and standard, 30 cycles were collected at 10 s integrations per scan. Between sample analyses, the system was washed with 10% HNO_3_ + 2% HCl to reduce the signal intensity for the CV system to the background level. The solution uptake rate was adjusted to 0.625 mL/min. The size of the bracketing standard was kept the same as that of the sample (0.5 ng/g). The typical intensity of ^202^Hg was ~ 0.8 V, and signal intensities observed for the blank samples were typically < 1% of those observed for the test samples.

#### Details of the procedure implemented at NIES

From each sample and standard, 50 cycles were collected at 10 s integrations per scan. Between sample analyses, the system was washed with 5% HCl to reduce the signal intensity for the CV system to the background level. The solution uptake rate was adjusted to 0.65 mL/min. The size of the bracketing standard was kept the same as that of the sample (1 ng/g). The typical intensity of ^202^Hg was ~ 0.6 V. Signal intensities observed for the blank samples were typically < 1% of those observed for the test samples.

The general settings used at IPREM and NIES are presented in Table 2.

**Table 2:** Settings of the cold vapor generation system coupled to a multicollector inductively coupled plasma mass spectrometry instrument

Instrumentation	*IPREM (Nu Plasma)*	*NIES (Nu Plasma II)*
Monitored isotopes*	198, 199, 200, 201, 202, 203, 204, 205, 206	198, 199, 200, 201, 202, 203, 204, 205, 206
Radio frequency power	1300 W	1300 W
Plasma gas	13.0 L/min	13.0 L/min
Auxiliary	0.80 L/min	0.80 L/min
Nebulization	1.0 L/min	1.0 L/min
Integration time	10 s	10 s
Sample uptake	0.625 mL/min	0.65 mL/min
Number of cycles per block	30 cycles/block	50 cycles/block
Number of blocks	1	1
Concentration (^202^Hg) of sample and standard	0.5 ng/g	1.0 ng/g
Intensity (^202^Hg) of sample and standard	~ 0.8 V	~ 0.6 V

*IPREM* Institut des Sciences Analytiques et de Physico-chimie pour l’Environnement et les Matériaux, *NIES* National Institute for Environmental Studies

*Atomic masses of 203 and 205 are those of Tl and 206 is that of Pb

The following raw isotopic ratios, ^199^Hg/^198^Hg, ^200^Hg/^198^Hg, ^201^Hg/^198^Hg, ^202^Hg/^198^Hg, and ^204^Hg/^198^Hg, were corrected for instrumental mass bias using the measured ^205^Tl/^203^Tl isotope ratios and its reference value (2.38714). The errors for these ratios were calculated by determining twice the standard deviation (2SD) of the sample and the bracketing standard measurement mean (2*σ*). Any ratio with a value greater than twice the population SD was rejected.

Generally, Hg isotope ratios are reported as actual ratios or *δ* values, which represent deviations in an isotope ratio in parts per thousand (denoted as ‰) from that of a standard. All sample analyses were bracketed by analysis of an Hg isotopic standard solution, NIST SRM 3133, and Hg isotopic ratios were calculated relative to the mean of the bracketing standards using the following equation [18]:

$$ {\delta}^{\ast \ast \ast}\mathrm{Hg}\left({\mbox{\fontencoding{U}\fontfamily{wasy}\selectfont\char104}} \right)=\left[{\left({}^{\ast \ast \ast}\mathrm{Hg}/{}^{198}\mathrm{Hg}\right)}_{\mathrm{sample}}/{\left({}^{\ast \ast \ast}\mathrm{Hg}/{}^{198}\mathrm{Hg}\right)}_{\mathrm{NISTSRM}3133}-1\right]\times 1000 $$

where *** represents one of the five other possible isotopic mass numbers for Hg (199, 200, 201, 202, and 204). In this study, the MIF factor is reported using the capital delta notation (Δ) as the difference between the measured δ^***^Hg and the same parameter’s theoretically predicted value using the following relationship:

$$ {\Delta}^{\ast \ast \ast}\mathrm{Hg}\left({\mbox{\fontencoding{U}\fontfamily{wasy}\selectfont\char104}} \right)={\delta}^{\ast \ast \ast}\mathrm{Hg}-\left(\beta \times {\delta}^{202}\mathrm{Hg}\right) $$

where *β* represents the equilibrium MDF factor, which is equal to 0.252, 0.502, 0.752, and 1.493 for ^199^Hg, ^200^Hg, ^201^Hg, and ^204^Hg, respectively [18].

### Total Hg mass fraction

A collaborative analysis for THg involving four organizations was undertaken: IPREM (AMA 254, ALTEC), NIPPON STEEL TECHNOLOGY Co., Ltd. (MA-2000, Nippon Instruments Co.), MURATA Keisokuki Service Co., Ltd. (SP-3D, Nippon Instruments Co.), and IDEA Consultants, Inc. (Hg-201, Sanso Seisakusho Co., Ltd.). The two bottles were sent to each organization (bottle numbers are shown in Table 3). Acid pretreatment using methods the Mercury Analysis Method [19] and Soil Analysis Method [20] from the Ministry of the Environment, Japan, were used by IDEA Consultants, Inc. and MURATA Keisokuki Service Co., Ltd., respectively, and a direct powder measurement was applied by IPREM and NIPPON STEEL TECHNOLOGY Co., Ltd.

**Table 3:** THg mass fraction of NIES CRM No. 28 Urban Aerosols

Organization	Instrumentation	Digestion	Bottle no.	Number of subsampling	Hg	2SD
					mg/kg	
IPREM	Thermal decomposition-atomic absorption spectrophotometry (TD-AAS), AMA254	direct powder measurement (no acid digestion)	066	3	1.22	0.03
			581	3	1.24	0.07
				Mean	1.23	0.05
NIPPON STEEL TECHNOLOGY Co., Ltd.	Thermal decomposition-atomic absorption spectrophotometry (TD-AAS), MA-2000	direct powder measurement (no acid digestion)	343	3	1.23	0.09
			847	3	1.25	0.05
				Mean	1.24	0.07
MURATA Keisokuki Service Co., Ltd.	Cold vapor-atomic absorption spectrophotometry (CV-AAS), SP-3D	HNO_3_-H_2_SO_4_-KMnO_4_	249	3	1.16	0.06
			985	3	1.17	0.03
				Mean	1.16	0.05
IDEA Consultants, Inc.	Cold vapor-atomic absorption spectrophotometry (CV-AAS), Hg-201	HNO_3_-HClO_4_-H_2_SO_4_	343	3	1.10	0.03
			847	3	1.14	0.10
				Mean	1.12	0.08
				Mean	2SD	2RSD%
			All	1.19	0.12	9.94

## Results

To describe data, two precision indicators, repeatability and reproducibility, are generally used. Repeatability represents variation that occurs when repeated measurements are made of the same item under absolutely identical conditions. Reproducibility represents variation that results when different conditions are used to make the measurements. The details are described in ISO 21748:2017 [21].

### Total Hg mass fraction

The repeatability and reproducibility of the THg mass fraction of NIES CRM No. 28 are reported in Table 3. After all of the data were combined, the THg mass fraction is determined to be 1.19 ± 0.12 mg/kg (2SD, *n* = 24) (Table 3). The THg mass fraction of the secondary reference, NIES CRM No. 33 (Landfill Cover Soil), was determined by three laboratories using the same methods. The reported value of the material was 0.31 mg/kg (http://www.nies.go.jp/labo/crm-e/Landfillcoversoil.html), while NIPPON STEEL TECHNOLOGY Co., Ltd., MURATA Keisokuki Service Co., Ltd., and IDEA Consultants, Inc. showed the THg of 0.32, 0.373, and 0.318 mg/kg, respectively.

### Hg isotopic compositions

NIST RM 8610 (UM Almaden) was used as a secondary standard and measured relative to NIST SRM 3133 several times within each analysis session. To report the analytical uncertainty of an unknown sample analysis, it is recommended to use an external reproducibility of the 2 standard error (SE) of replicate analyses unless it is smaller than the 2SD external reproducibility of the method using the in-house secondary standard [18]. In this study, the 2SD values of NIST RM 8610 (Table 4) were used for the analytical uncertainty of the measurement. To validate the analytical stability of our operating conditions, the repeatability of the Hg isotopic compositions of the secondary reference standard, NIST RM 8610, adjusted to the value of 0.5 and 1.0 ng/mL at IPREM and NIES, respectively, was monitored during the study period (Table 4). Drifting of Hg isotopic ratios may occur during a day-long analysis because of Ar gas flow instability, cone and slit degradation, and/or cup aging. To overcome these potential problems, all sample analyses were bracketed by the results of the analysis of the relevant standard, NIST SRM 3133, and the Hg isotopic values of the sample were calculated relative to the mean values of the corresponding parameters for the bracketing standard. Applying the standard-sample bracketing method, the deviations of the isotopic ratios measured for NIST RM 8610 were < 0.3‰ (*n* = 15), in the case of 0.5 ng/mL solutions, and our results showed in agreement with published data of Estrade et al. [22]. Hg isotopic measurements of a secondary reference material, BCR-176R, were also performed using the same dissolution and measurement methods (Table 4). Note that BCR-176R was analyzed at least twice on different days to monitor instrument stability. According to these measurements, the values for δ^202^Hg were − 1.05 ± 0.10‰ (*n* = 4), − 1.23 ± 0.10‰ (*n* = 4) and − 1.07 ± 0.15‰ (*n* = 4), and those for Δ^199^Hg were − 0.09 ± 0.08‰ (*n* = 4), − 0.10 ± 0.09‰ (*n* = 4) and − 0.07 ± 0.07‰ (*n* = 4) using digestion methods of HotBlock®, microwave, and digestion bomb, respectively. These values were identical (within an acceptable error) to their literature counterparts (δ^202^Hg = − 1.03 ± 0.15‰, Δ^199^Hg = − 0.06 ± 0.07‰, *n* = 8, Estrade et al. [17]).

**Table 4:** Hg isotopic compositions of NIST RM 8610 and BCR-176R

Sample	References		δ^199^Hg	δ^200^Hg	δ^201^Hg	δ^202^Hg	δ^204^Hg	Δ^199^Hg	Δ^200^Hg	Δ^201^Hg	Δ^204^Hg
			‰	‰	‰	‰	‰	‰	‰	‰	‰
NIST RM 8610 (UM Almaden)	IPREM	Mean	− 0.17	− 0.28	− 0.45	− 0.55	− 0.81	− 0.02	0.01	− 0.03	− 0.01
	*n* = 15	2SD	0.06	0.10	0.16	0.20	0.31	0.06	0.10	0.07	0.14
	NIES	Mean	− 0.11	− 0.22	− 0.40	− 0.49	− 0.73	0.02	0.03	− 0.03	0.01
	*n* = 4	2SD	0.08	0.11	0.17	0.10	0.07	0.07	0.09	0.10	0.13
	Estrade et al., [22]	Mean	− 0.18	− 0.32	− 0.48	− 0.61	− 0.94	− 0.03	− 0.02	− 0.02	− 0.03
	*n* = 5	2SD	0.03	0.01	0.07	0.14	0.23	0.02	0.06	0.03	0.10
BCR-176R (Fly Ash)	IPREM (HotBlock®)	Mean	− 0.36	− 0.53	− 0.90	− 1.05	− 1.57	− 0.09	− 0.01	− 0.12	− 0.01
	*n* = 4	2SD	0.10	0.07	0.14	0.10	0.19	0.08	0.08	0.08	0.14
	IPREM (microwave)	Mean	− 0.41	− 0.62	− 1.00	− 1.23	− 1.82	− 0.10	0.00	− 0.07	0.01
	*n* = 4	2SD	0.12	0.12	0.11	0.10	0.34	0.09	0.07	0.05	0.30
	NIES (digestion bomb)	Mean	− 0.34	− 0.53	− 0.86	− 1.07	− 1.61	− 0.07	0.00	− 0.06	− 0.02
	*n* = 4	2SD	0.10	0.09	0.13	0.15	0.12	0.07	0.02	0.03	0.12
	Estrade et al., [17]	Mean	− 0.32	− 0.51	− 0.83	− 1.03		− 0.06	0.00	− 0.06	
	*n* = 8	2SD	0.09	0.10	0.11	0.15		0.07	0.06	0.04	

**Fig. 1:**
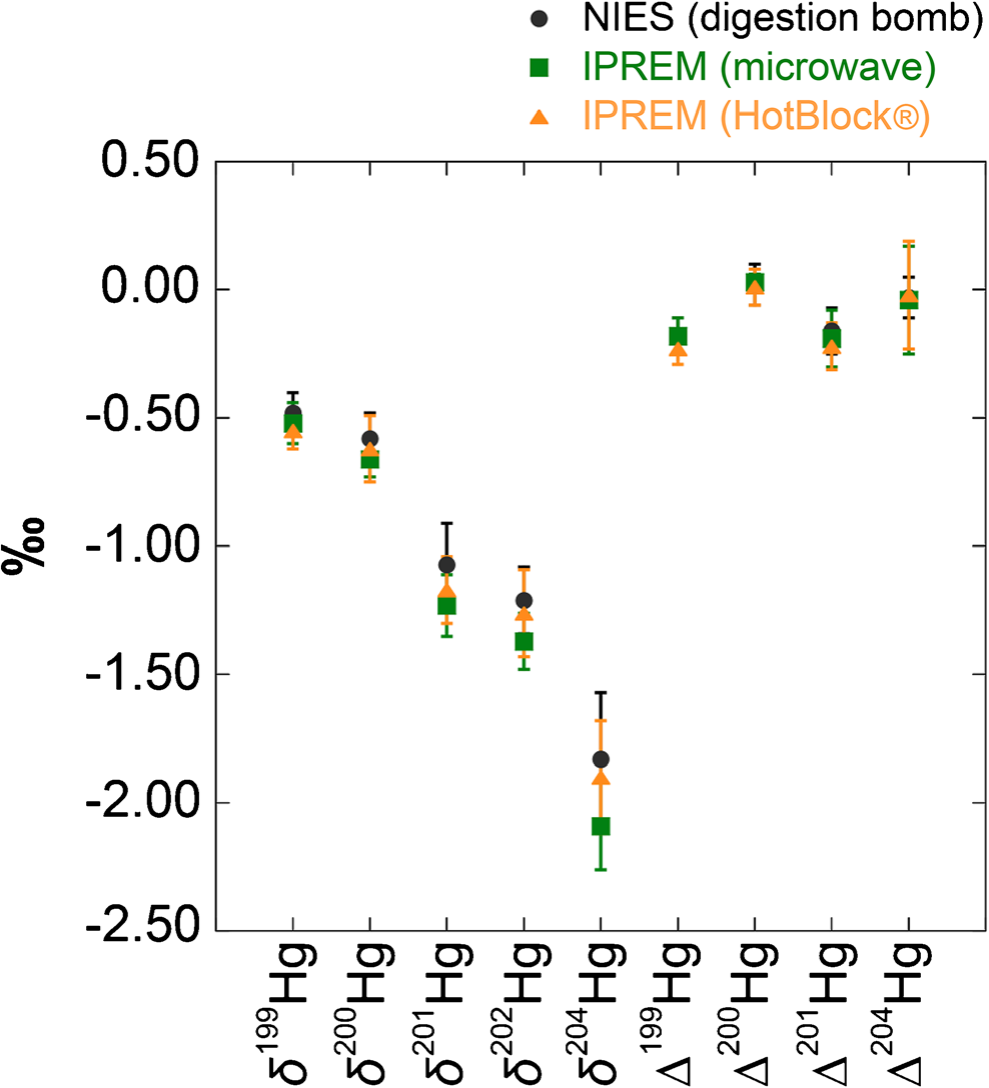
Hg isotopic compositions of NIES CRM No. 28. Three digestion methods were implemented in this study (digestion bomb: black circle; microwave: green square; and HotBlock®: orange triangle). The error bars on the CRM represent 2SD of Hg isotope heterogeneity from Table 5. IPREM, Institut Pluridisciplinaire de Recherche sur l’Environnement et les Matériaux; NIES, National Institute for Environmental Studies

NIES CRM No. 28 was digested using three different methods (see Electronic Supplementary Material (ESM) Table S1). Based on the CRM’s THg value, recovery yields measured after implementation of the three digestion methods were ~ 80%, ~ 90%, and ~ 100% for digestion bomb, microwave, and HotBlock®, respectively. Our repeated measurements showed δ^199^Hg = − 0.55 ± 0.07‰, δ^200^Hg = − 0.62 ± 0.13‰, δ^201^Hg = − 1.17 ± 0.13‰, δ^202^Hg = − 1.26 ± 0.17‰, and δ^204^Hg = − 1.90 ± 0.22‰, and Δ^199^Hg = − 0.23 ± 0.06‰, Δ^200^Hg = 0.01 ± 0.07‰, Δ^201^Hg = − 0.22 ± 0.09‰, and Δ^204^Hg = − 0.02 ± 0.21‰ (2SD, *n* = 18) for samples using HotBlock® (Table 5). The uncertainty of the Hg isotopic values is an expanded uncertainty determined using a coverage factor *k* = 2, which corresponds to the confidence interval of ~ 95%; the type B uncertainty and uncertainty in the bias of the methods are not included. The possibilities of the lower recovery yields for digestion bomb and microwave are incomplete dissolution or Hg loss with sorption or evaporation. Even if those may have happened, all analysis results across methods were consistent with each other, and variations were reported to be equivalent to those of the repeated measurements of NIST RM 8610 (Table 4 and Fig. 1).

**Table 5:** Hg isotopic compositions of subsamples of NIES CRM No. 28 Urban Aerosols measured using different sample digestion methods

Instrumentation	Digestion	Bottle no.	Number of subsampling	Number of measurements for each subsample	δ^199^Hg	2SD	δ^200^Hg	2SD	δ^201^Hg	2SD	δ^202^Hg	2SD	δ^204^Hg	2SD	Δ^199^Hg	2SD	Δ^200^Hg	2SD	Δ^201^Hg	2SD	Δ^204^Hg	2SD
					‰	‰	‰	‰	‰	‰	‰	‰	‰	‰	‰	‰	‰	‰	‰	‰	‰	‰
Nu Plasma	HotBlock®	581	3	2	− 0.58	0.07	− 0.64	0.12	− 1.23	0.13	− 1.30	0.17	− 1.97	0.33	− 0.25	0.05	0.01	0.04	− 0.25	0.05	− 0.03	0.22
		901	3	2	− 0.53	0.06	− 0.59	0.10	− 1.12	0.08	− 1.23	0.17	− 1.82	0.23	− 0.22	0.06	0.03	0.07	− 0.19	0.13	0.01	0.16
		990	3	2	− 0.54	0.09	− 0.63	0.19	− 1.15	0.17	− 1.26	0.16	− 1.91	0.10	− 0.22	0.06	0.01	0.11	− 0.21	0.07	− 0.03	0.26
				Mean	− 0.55	0.07	− 0.62	0.13	− 1.17	0.13	− 1.26	0.17	− 1.90	0.22	− 0.23	0.06	0.01	0.07	− 0.22	0.09	− 0.02	0.21
Nu Plasma	microwave	581	3	2	− 0.54	0.06	− 0.68	0.07	− 1.26	0.16	− 1.42	0.08	− 2.09	0.19	− 0.19	0.05	0.04	0.03	− 0.20	0.13	0.02	0.24
		990	3	2	− 0.51	0.09	− 0.65	0.07	− 1.19	0.09	− 1.33	0.14	− 2.09	0.15	− 0.17	0.08	0.02	0.03	− 0.19	0.08	− 0.11	0.19
				Mean	− 0.52	0.08	− 0.66	0.07	− 1.23	0.12	− 1.37	0.11	− 2.09	0.17	− 0.18	0.07	0.03	0.03	− 0.19	0.11	− 0.04	0.21
Nu Plasma II	digestion bomb	960	3	2	− 0.48	0.08	− 0.58	0.10	− 1.07	0.16	− 1.21	0.13	− 1.83	0.26	− 0.18	0.07	0.02	0.08	− 0.16	0.09	− 0.03	0.08

## Discussion

### Homogeneity

The δ^202^Hg values using HotBlock® were tested by one-way analysis of variance (ANOVA) to investigate the homogeneity of isotopic results in the CRM (Table 6). The between-bottle variation evaluated by one-way ANOVA did not show any statistically significant difference (*p* > 0.05 and *F*_calculated value_ < *F*_critical value_). Therefore, the CRM is homogeneous between 0.05 and 0.3 g when applied to the Hg isotopic values, as presented in this paper.

**Table 6:** ANOVA data from the homogeneity study for the Hg isotope

	*F* value	*p* value	*F* critical value	*S*_bb_	*U*_bb_
Hg isotope (δ^202^Hg)	1.067	0.3687	3.682	0.7%	2.1%

*S*_*bb*_ between-bottle variance, *U*_*bb*_ between-bottle variance incorporating the influence of analytical variation

### Sample digestion for Hg isotopic measurement

To test the bias of sample digestion methods, we applied three digestion methods. Using the preliminary data of the THg mass fraction of the CRM, the recovery yields were higher in HotBlock®, followed by those in a microwave and in a digestion bomb. This observation may be attributed to an insufficient sample dissolution in a microwave and a digestion bomb. To ensure the complete recovery of the sample, a mixture of HNO_3_/HCl/HF is generally used for the elemental analysis of geological samples (e.g., soils and sediments). HF is essential in dissolving silica matrices via the reaction HF + SiO_2_ → H_2_SiF_6_ + H_2_O. After the sample decomposition using HF, HF must be evaporated by heating before analysis. However, Hg will be partially evaporated during heating, which may result in Hg isotope fractionation. Therefore, HF was not used for most Hg studies, and concentrated HNO_3_ combined with HCl [23] or H_2_SO_4_ [22] was used to determine the THg mass fraction. The decomposition method using HNO_3_/HCl/H_2_O_2_ with HotBlock® was also applied for an extended period of time to perform the Hg isotopic analysis of soil and sediment [15, 16]. Another possibility for lowering Hg concentration may be Hg loss with sorption or evaporation during acid decomposition using digestion bomb and microwave. Hg loss may also occur during the preservation of dissolved samples,but the digested samples were stored, without dilution, in a refrigerator before measurements. Despite the incomplete Hg dissolution or the Hg loss during/after bomb and microwave digestions, the analysis results for all methods used here were consistent with each other, and the variations were reported to be equivalent to those of the imprecision of the CV-MC-ICP-MS measurement. Thus, NIES CRM No. 28 is isotopically homogeneous for subsamples of weights ranging between 0.05 and 0.3 g.

### Comparisons between NIES CRM No. 28 and plausible Hg emission sources, and aerosols collected in China

**Fig. 2:**
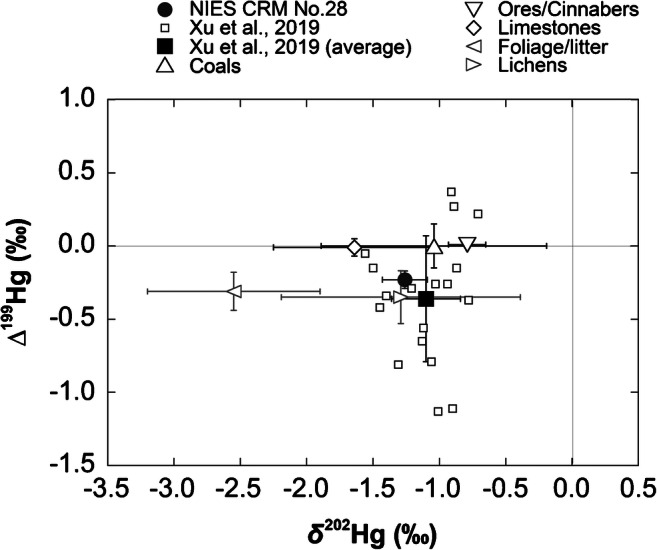
Values for Δ^201^Hg versus those for Δ^199^Hg measured for NIES CRM No. 28 (filled black circle), PM2.5 from Beijing (open black squares for individual data and filled black square for the average), and plausible source materials (coals: open black triangle; ores/cinnabars: open black inverted triangle; limestones: open black diamond; foliage/litter: open black left-pointing triangle; and lichens: open black right-pointing triangle). The error bars on source materials and PM2.5 sample average represent SD of Hg isotope heterogeneity, while NIES CRM No. 28 represent 2SD

According to previous reports [24–26], coal combustion, non-ferrous metal smelting, and cement production were major emission sources of Hg found in PMs in China during the period when the CRM was collected (1995–2005). The Hg isotopic compositions of NIES CRM No. 28, Chinese coals [11], non-ferrous metal ores [27], limestones (for cement production) [28], and cinnabars (for industrial uses) [29] are shown in Fig. 2. Hg isotopic compositions of PM2.5 samples analyzed at the Chinese Academy of Science in Beijing were previously reported. In particular, Xu et al. [12] collected PM2.5 samples from December 2013 to January 2014 and found the average values of δ^202^Hg and Δ^199^Hg to be − 1.10 ± 0.26‰ and − 0.36 ± 0.43‰ (1σ, *n* = 18), respectively. The δ^202^Hg and Δ^199^Hg values for the CRM overlap with the previously reported Hg isotopic variation ranges (Fig. 2). Previously, studies demonstrated that industrial or combustion processing of source materials causes significant MDF, but not for MIF [11, 29–32]. The major Hg emission source(s) might not have substantially changed during the sampling period in the study area. Samples derived from ores, limestones, and cinnabars were characterized by negative δ^202^Hg values and by Δ^199^Hg values of ~ 0. Because the CRM and Beijing PM2.5 were characterized by negative Δ^199^Hg values, results from this study may point to the existence of additional, different Hg emission sources (e.g., the biomass burning of foliate/litter and lichens, wood fire heating and cooking).

## Conclusions

NIES CRM No. 28 Urban Aerosols was originally prepared for certifying the mass fractions of major and minor elements. In this study, the Hg isotopic composition of the CRM was determined to provide an appropriate quality assurance/quality control tool for the Hg isotopic analysis of PMs. To validate and ensure the accuracy of our method, analytical uncertainty was estimated based on replication of the NIST RM 8610 (UM Almaden) standard solution. According to our results with respect to within- and between-bottle variations of subsamples of the CRM using a conventional dissolution method using a HNO_3_/HCl/H_2_O_2_ mixture with HotBlock®, the CRM is sufficiently homogenous to be used in Hg isotopic measurements. Two different digestion methods were applied in this study. Although two other methods showed a lower Hg recovery yield than that of the conventional method, all Hg isotopic compositions were equivalent. Our isotopic analysis results may contribute to quality assurance in environmental monitoring studies of aerosols.

## Electronic supplementary material

ESM 1 (PDF 78 kb)
