# Supplementary material for: Hg isotopic composition and total Hg mass fraction in NIES Certified Reference Material No. 28 Urban Aerosols
Source: Anal Bioanal Chem. 2020 May 18;412(19):4483–93. doi: 10.1007/s00216-020-02691-9 (PMC7329778; doi:10.1007/s00216-020-02691-9)
Supplement: Supplementary file 1 — (PDF 78 kb) [file 216_2020_2691_MOESM1_ESM.pdf]

Analytical and Bioanalytical Chemistry

Electronic Supplementary Material

Hg isotopic composition and total Hg mass fraction in NIES Certified Reference Material No. 28 Urban Aerosols

Akane YAMAKAWA, Sylvain BÉRAIL, David AMOUROUX, Emmanuel TESSIER, Julien BARRE, Tomoharu SANO, Kimiyo NAGANO, Sadia KANWAL, Jun YOSHINAGA, Olivier F. X. DONARD

Table S1 Methods implemented to treat the certified reference material to assess its Hg isotopic homogeneity

	Pretreatment	CV-MC-ICPMS instrument
A	Sample heated at 85°C for 24 h in the presence of HNO_3, HCl, and H_2O_2 (v/v 3:1:1) in a PP tube and PE cap using HotBlock®, closed system	Nu Plasma at IPREM
B	Sample heated at 230°C for 25 min in the presence of HNO_3 and HCl (v/v 3:1) in a glass tube and Teflon cap using a microwave chamber, closed system	Nu Plasma at IPREM
C	Sample heated at 130°C for 3 h in the presence of HNO_3 and HCl (v/v 1:3) in a Teflon capsule using a digestion bomb, closed system	Nu Plasma II at NIES

CV-MC-ICPMS: cold vapor generation coupled to multi-collector inductively coupled plasma mass spectrometry; IPREM: Institut des Sciences Analytiques et de Physico-chimie pour l'Environnement et les Matériaux; NIES: National Institute for Environmental Studies.
